# Prevalence and factors associated with intestinal parasites among children of age 6 to 59 months in, Boricha district, South Ethiopia, in 2018

**DOI:** 10.1186/s12887-020-1935-3

**Published:** 2020-01-22

**Authors:** Berhan Tsegaye, Amanuel Yoseph, Hunachew Beyene

**Affiliations:** 1 Department of Midwifery, college of medicine and health sciences, Hawassa University, Hawassa, Ethiopia; 2 Department of public health, college of medicine and health sciences, Hawassa University, Hawassa, Ethiopia; 3 Department of Environmental, College of Medicine and Health Sciences, Hawassa University, Hawassa, Ethiopia

**Keywords:** Intestinal parasites, Children, 6 to 59 months, Ethiopia

## Abstract

**Background:**

Intestinal parasites are the commonest cause of childhood diarrhea and malnutrition in Ethiopia. Information about intestinal parasites is the first fundamental step for designing intervention strategies against them. Hence, health planners can maximize their efforts. Information is scarce about intestinal parasites among children of under-five years of age in Boricha district. Therefore, this study aimed at assessing prevalence and factors associated with intestinal parasites among children of age 6 to 59 months in Boricha district, South Ethiopia.

**Methods:**

A community based analytical cross sectional study was conducted among 624 children of age 6 to 59 months from January 1 to 30; in 2018. We have utilized two stage stratified sampling method. Firstly, simple random sampling was used to select sample Kebeles. Secondly, systematic random sampling method was used to select the study participants. Structured and interviewer administrated questionnaire was used to collect data. Parasitological examination of children’s stool was conducted microscopically. Data were entered into Epi-info, exported and analyzed by SPSS version 22. Logistic regression analysis was conducted to identify association between explanatory variables and outcome variable. Adjusted Odds Ratio (AOR) with 95% confidence interval (95%CI) was computed, and *P*-value < 0.05 was considered as statistically significant. Descriptive statistics was presented using texts, tables and figures.

**Result:**

A total of 622 participants were included in the analysis which makes a response rate of 99.9%. Prevalence of intestinal parasites was 48.7% (95%CI, 44.8–52.6) in this study. Higher family size (AOR = 2.7, 95%CI = 1.5–5.0), medium family size (AOR = 2.3,95%CI,1.3–4.2), absence of laterine facility in the household (AOR = 2.9, 95% CI = 1.6–5.3), unable to put on shoes (AOR = 3.5,95%CI = 2.2–5.7), and eating raw vegetables (AOR = 2.6,95%CI = 1.6–4.7) were factors positively associated with intestinal parasites in this study.

**Conclusion:**

Overall prevalence of intestinal parasites was almost high. Latrine facility, family size, shoes wearing habit and eating raw vegetables were significantly associated with intestinal parasites. Family planning service, sanitation and hygiene practices should be intensified through community education. Activate support of deworming program should be considered. Moreover, policy makers should give priority on creating awareness to prevent intestinal parasite.

## Background

Intestinal parasites are inhabitants of gastro-intestinal tract. They can cause malnutrition on different population groups. Children and poor people are the most vulnerable groups since they have more chance of contact with contaminated soil in their daily life [1]. Poor personal hygiene and lack of clean water are the main causes of intestinal parasites. Intestinal parasites could exist predominantly in polluted, overcrowded and dirty places [2]. Intestinal parasites are manifested by diarrhea, abdominal cramp, vomiting and weight loss. These symptoms can be more severe among children, under nourished and immune compromised patients than other group of population [3–5]. Diarrhea is a predominant consequence of intestinal parasites. It is the leading cause of mortality and morbidity of under five children [6–8]. In addition, it can aggravate protein energy malnutrition, anemia and other nutrient deficiencies [9]. Moreover, intestinal parasites have been associated with short and long term complications on children. These include the following health complications: stunting, physical weakness, insufficient educational achievement, poor reproductive health, and low economic development [10, 11].

Intestinal parasites are the most prevalent infections among children of poor and rural families since they have contacts with soil on their farm in most of the time. So, children are more prone for infective larvae through direct or indirect transmission route [1]. Children of under-five years of age are the most vulnerable group due to their low immunity status. Hence, they need special care and follow up [12]. Despite the existence of different prevention strategies like, chemotherapy and health promotion, intestinal parasites are a widely spread problem in the world [13]. According to Ethiopia Schistosomiasis and soil-transmitted Helminthes control program report, there is a short term target of reduction of the current heavy and moderate soil transmitted helminthic infection intensity to less than 1% at the end of the program in Ethiopia [14]. But, a study conducted in Babile town, Eastern Ethiopia shows that prevalence of intestinal helminthes is 27.2% [15]. *A. lumbricoides* and *T. trichiura* are the commonest and widely distributed parasites throughout Ethiopia [16]. Besides, protozoa are the other dominant infections which exert huge impact in Ethiopia [4].

When prevalence of intestinal parasites is less than 20%, it is considered as lowest. Moreover, it is considered as moderate when it is from 20 to 50%. It is also classified as heavy when the prevalence is greater than 50% [17]. The long term global target is to eliminate mortality and morbidity associated with intestinal parasites among children by 2020 [18]. According to Boricha health office report, intestinal parasites affect almost all Kebeles of the district. Preventive and curative actions were performed. Among these, all school age children were dewormed every two to five years regularly. The commonest type of toilet was a pit latrine without a slab or open pit in the district [19]. Farming activity is the main source of income in Boricha district; inhabitants of district mainly produce Enset, cash crops (khat, coffee) and livestock. Unclean drinking water source, poor hygienic condition and frequent drought in the district results high burden of the under-nutrition and intestinal parasites [20]. Poor hygiene related diseases, like acute watery diarrhea and intestinal parasites, are among the leading causes of childhood morbidity and mortality [19]. Although many studies have been conducted about intestinal parasites in Ethiopia [21, 22]. Information is scarce in the current study area about intestinal parasites among children of age 6 to 59 months. Moreover, most previous studies were focused on school aged children but studies were limited among children aged 5 to 59 months. Therefore, the main aim of this study is to assess prevalence and factors associated with intestinal parasite infections among children of 6 to 59 months in Boricha district, South Ethiopia, in 2018.

## Methods

### Study area

The study was carried out at Boricha district, Sidama zone, South Ethiopia. It is located 206 km from Addis Ababa, the capital city of Ethiopia, and 32 km from Hawassa main city of Southern nation nationalities and peoples of Ethiopia. According to the 2018 Ethiopian central statistical agency report, the total population of the district was estimated to be 325,636. From these, nearly all (97%) population live in rural area. Children of 6 to 59 months’ age account of 13.94% of the population. Boricha consists of 4 urban and 39 rural Kebeles. The health service coverage of the district was 85%. It has one government district hospital, ten health centers, thirty-nine health posts and eight private clinics.

### Study design, period and population

Community based cross sectional study was carried out in Boricha district from January 1st to 30th, 2018. The source population were all children aged 6 to 59 months who live in Boricha district. The study population were children aged 6 to 59 months who lived in randomly selected household. Those children whose family lived less than 6 months, took preventive chemotherapy (deworming), and treated recently for intestinal parasites were excluded from this study.

### Sample size determination and sampling technique

Sample size was calculated by using single population proportion formula in consideration of the following assumptions: The prevalence of intestinal parasites was taken as 24.3% [23], 95% confidence level, 5% margin of error. Thus, n = z^2^pq/d^2^. Then, *n* = 1.96^2^*0.243(0.757)/0.05 = 283. By using a design effect of 2 and adding 10% non-response rate, a total sample sizes of 624 were included. Boricha was selected intentionally by considering its context of exposure for intestinal parasites and lack of previous research related to the problem. There are 43 Kebeles in the district. Of these, 4 were urban and 39 were rural Kebeles. A two stage sampling technique was applied to select the representative sample size of this study. Firstly, eight Kebeles were selected using simple random sampling based on their proportion of place of residence which satisfy 20–30% of the total district Kebeles. Secondly, a sampling frame was prepared through census. It consisted of lists of household in the selected Kebeles. There were a total of 8482 children in the selected Kebeles. The calculated sample interval (K=N/n) was 14. Then, systematic random sampling strategy was used to select the study participants. The first child was identified by using simple random sampling strategy by using lottery method. Then, consecutive children were selected at a regular interval of 14th household. If there was no child from the household for consecutive visits, the next nearest child was included. One child was included by using lottery method when more than one child was present selected households. Each child was given a unique identifier to be identified during the socio-demographic and stool examination.

### Study variables

In the current study, the outcome variable was presence of intestinal parasites. It is a binary outcome variable. Independent variables included socio-demographic, environmental and health seeking behavior variables. Socio-demographic variables included the following variables: age and sex of child, religion, ethnicity, family size, wealth index, maternal/paternal education and occupation, marital status and media availability. Environmental variables consisted of variables like, water source, availability of latrine, availability of safe excreta disposal, type of house and numbers of room. Health-seeking behavior variables included the following variables: personal hygiene practice, type of water consumption, water handling technique, hand washing practice, playing with soil, habit of eating raw vegetables and fruits and wearing shoes, health service availability, household food insecurity status and dietary diversity score.

#### Operational definition

##### Intestinal parasite

Is present when a child is infected with at least one intestinal parasite infection.

##### Family size

Family size is defined as a total number of family members living in the household. Family size is classified as small when it is less than six. It is classified as medium when it is between six to eight, and it is classified as large when it is greater than eight.

### Data collection method and tools

Data were collected by face to face interview using a structured and pre-tested questionnaire. Data were collected in Sidama language from children’s parents (caregivers). Interviewers have also inspected whether children wore shoe or not. Data were collected by eight diploma nurses, two laboratory technicians, and eight community health workers. Two health officers supervised data collection process.

### Stool collection and examination

All parents / guardians/ were informed by health extension workers to bring their children of age 6 to 59 months to health posts. Parents or guardians were asked to provide about 2 g of fresh stool sample of their children using clean, tightly corked, leak proof containers. The sample was analyzed using wet mount technique and the remaining portion was concentrated using formal ether concentration technique [24]. The specimens were examined microscopically for the presence of eggs, trophozoites or cysts. Stool samples were collected from 622 children whose age was between 6 to 59 months for intestinal parasites examination.

### Data quality control

Questionnaire was prepared in English language, and translated into Sidama language (local language). Then, it was re-translated back to English to keep its consistency. Comparison was done between two versions to assess inconsistent and inaccurate data. It was pre-tested by 5% of sample size in Kebeles which were not included in the actual study area. Finally, any inconsistent and inaccurate data was re-adjusted accordingly. Data collectors and supervisors were given two-day training before the actual data collection time. The training was focused on the aim, procedure and data collection technique.

### Data processing and analysis

Data were cleaned, coded and entered in to a computer using Epi data. Then, data were exported and analyzed by SPSS version 22. Descriptive statistics was used to show prevalence of intestinal parasites. Tables and graphs were formed to present data. Binary logistic analysis was done first and variables with *p*-value < 0.25 were considered as candidate for multivariable logistic regression. Model fitness was checked using Hosmer-Lemeshow test of goodness of fit before the actual logistic regression analysis. Adjusted Odds Ratio (AOR) with 95% confidence interval (CI was reported. Variables which have *P*-value of less than 0.05 in the multivariable logistic regression analysis was considered as significantly associated factors of intestinal parasites. Finally, those variables whose *p*-value less than 0.05 were considered as statistically significant in multi-variable logistic regression.

## Results

Table 1 presents socio-demographic characteristics of study subjects. From a total of 624 children who participate in this study, children who gave full response were 622 making a response rate of 99.9%. Mean age of children was 29.25 months. Nearly 510 (82%) of households got their income from farming activities. Four-hundred- one (64.4%) and three-hundred- four (63.3%) of the parents never attended formal education. The studied households had a median family size of approximately six persons. Almost all of the households 614(98.7%) were headed by males. The two hundred-forty-two (38.9%) of the households had access to mass media (insert Table.1).

**Table 1:** Socio-demographic characteristics of study participants in Boricha district, South Ethiopia, 2018 (*n* = 622)

Variables	Categories	Number (%)
Sex of child	Male	305 (49%)
	Female	317 (51%)
Age of child	6–11 months	105 (24.5%)
	12–23 months	126 (20.3%)
	24–59 months	343 (55.2%)
Ethnicity	Sidama	596 (95.8%)
	Others	26 (4.2%)
Religions	Protestant	517 (83.1%)
	Others	105 (16.9%)
Family size	Small	300 (48.2%)
	Medium	117 (18.8%)
	Large	205 (33%)
Maternal educational status	No formal education	401 (64.4%)
	Primary education	159 (25.6)
	Secondary education & above	62 (10%)
Paternal educational status	No formal education	394 (63.3%)
	Primary education	122 (19.6%)
	Secondary education & above	106 (17.1)
The main occupation of the head	Farmer	505 (81.2%)
	Daily worker	117 (18.8%)
Wealth Index	Poorest	154 (24.8%)
	Poor	72 (11.6%)
	Middle	157 (25.2%)
	Rich	117 (18.8%)
	Richest	122 (19.6%)

Households which got protected water sources were 485 (78%). The majorities (67.5%) of the study households had pit latrines without a slab. Two-hundred fifty-two (40.4%) of children did not wear shoes. The majorities (65.8%) of households used a single room to sleep (insert Table.2).

**Table 2:** The sanitation and personal hygiene condition of studied children aged 6–59 months, Boricha District, Southern Ethiopia, 2018 (*n* = 622)

Variable	Categories	No (%)
The main source of drinking water	Protected	485 (78%)
	Unprotected	137 (22%)
Availability of toilet facility	yes	449 (72.2%)
	No	173 (27.8%)
Types of latrine	Pit latrine without slab	420 (67.5%)
	Pit latrine with slab	29 (4.7%)
Number of rooms used to sleep	One	408 (65.8%)
	Two	214 (34.2%)
Hand washing practice	Yes	394 (63.3%)
	no	228 (36.7%)
Shoes wearing habit	Yes	371 (`59.6%)
	No	251 (40.4%)
Practice of eating raw vegetable	Yes	184 (29.6%)
	No	438 (70.4%)

### Prevalence of intestinal parasites among children of age 6 to 59 months

*Giardiasis* 255(41%) and *ascariasis* 280(45%) were common parasites among females. However, *trichuriasis 180*(27%) and *hookworm* 205(33%) were common among males. The overall prevalence of at least one intestinal parasite was 48.7% (95%CI, 44.8–52.6). The prevalence of protozoa was, *Giardia lamblia* 65(10.45%) and *Entamoeba histoltica* 29(4.66%). In addition, helminthes were, A. *lumbricoides* 67 (10.77%), *Hookworm* 49 (7.88%), *T. trichiura* 38 (6.1%), *S. stercoralis* 10 (1.6%) and *Tinea species* 8 (1.3%). Mixed infections accounted for 37 (5.94%) of children (insert Table.3).

**Table 3:** prevalence of intestinal parasites among children of age 6–59 months in Boricha district, Southern Ethiopia, 2018 (*n* = 622)

Variable	Categories	No (%)
Types of infections	Single	266 (42.8%)
	Mixed	37 (5.9%)
Types of intestinal parasites	*Giardia lamblia*	65 (10.45%)
	*Entamoeba histoltica*	29 (4.66%)
	*Ascaris lumbricoides*	67 (10.77%)
	*Trichiuris trichiuria*	38 (6.1%)
	*Hookworm*	49 (7.88%)
	*Tinea species*	8 (1.3%)
	*Strongliod stercorias*	10 (1.6%)

### Determinants of intestinal parasites

Results of multivariable logistic regression analysis are shown in Table 4. Accordingly, wealth index, main source of drinking water, eating raw vegetables, absence of latrine, wearing shoe and family size were factors significantly associated with intestinal parasites. Children from large family size (more than eight family member) had 2.7 fold of higher odds of intestinal parasites than children from lower family size (AOR = 2.70; 95% CI = 1.5–5.0, *P* = 0.001). In addition, children from medium family size (six to eight) had 2.3 more chance of infection than children of lower family size (AOR = 2.3,95%CI,1.3–4.2, *p* = 0.03). The likelihood of getting intestinal parasites among children who had a habit of eating raw vegetables was 2.6 times higher than their counterparts (AOR = 2.65, 95 CI = 1.60–4.70, *P* = 0.002). The odds of infection by intestinal parasites among children living in the household with absence of latrine were 2.9 times higher than their counterparts (AOR = 2.9; 95% CI = 1.60–5.30, *P* = 0.001). Unable to wore shoes, in contrast to wearing shoes showed an increased odds of infection by intestinal parasites (AOR = 3.5; 95% CI =2.20–5.70, P = 0.001) (insert Table.4).

**Table 4:** Determinant of intestinal parasitic infections from backward stepwise (Wald) logistic regression in among children aged 6 to 59 months, Boricha district, South Ethiopia, 2018 (*n* = 622)

Variable	Categories	Intestinal parasites		COR (95% CI)	AOR(95% CI)	*p*-value
		Yes	No			
Wealth index	Poorest	113	41	5.65 (3.35,9.5)*	1.04 (0.49,2.22)	0.62
	Poor	39	33	2.42 (1.33,4.4)*	0.79 (0.34,1.78)	0.57
	Middle	69	88	1.6 (0.98,2.62)	0.57 (0.29,1.10)	0.72
	Rich	42	75	1.14 (0.67,1.95)	0.57 (0.28,1.15)	0.64
	Richest	40	82	1	1	
Source of drinking water	Protected	199	286	1	1	
	Unprotected	104	33	4.52 (3.13,7.62)*	1.14 (0.58,2.24)	0.351
Raw vegetables & fruits	Yes	143	41	6.0 (4.07,9.02)*	2.65 (1.6,4.7)**	0.002
	No	160	278	1	1	
Absence of latrine	Yes	135	38	5.9 (4,8.9)*	2.9 (1.6,5.3)**	0.001
	No	168	281	1	1	
Wearing Shoes	Yes	127	244	1	1	
	No	176	75	4.5 (3.2,6.4)*	3.5 (2.2,5.7)**	0.001
Family size	Small	78	222	1	1	
	Medium	71	46	4.4 (2.8,6.9)*	2.3 (1.3,4.2)**	0.03
	Large family	154	51	8.6 (5.7,12.9)*	2.7 (1.5,5)**	0.001

Key: 1 = reference categories; * = Indicates significant association (*P*-value < 0.25); ** = Indicate the highly significant association (*P*-value < 0.05); *COR* Crude Odds Ratio, *AOR* Adjusted Odds Ratio, *CI* Confidence Interval

## Discussion

Prevalence of intestinal parasite infection was 48.7% (95%CI, 44.8–52.6) in this study. Larger family size, medium family size, absence of laterine, unable to put on shoe, eating raw vegetable were factors positively associated with intestinal parasites. When the prevalence of intestinal parasite was reached to 50% and above, it is considered as high in Ethiopia. According to national classification of intestinal parasite prevalence, the finding of the current study is reached to high category [17]. In addition, it is far from short term national target reduction of intestinal parasite of less than 1% in Ethiopia. So, it is impossible to meet national target of eliminating parasites in 2020 [14]. This finding is consistent with a study conducted in Hawassa Zaria,South Ethiopia (51.3%) and Senbete town,North Shoa,Ethiopia (52.3%) [25, 26]. The possible explanation might be due to similarities of certain characteristics parents of children in the current study and studies mentioned above. These include the following factors: socio-economic status, level of awareness about the problem and life styles. Besides, it may be due to similar study population in these studies. This finding is higher than findings of studies conducted in Wonji Shoa Sugar Estate, Ethiopia (24.3%) [23] and Hawassa, South Ethiopia (26.6%) [27]. This might be explained by a number of factors. They were low socio-economic condition, specific climate, poor sanitary and hygiene condition in the current study area. On the contrary,this finding is lower than finding of a study conducted in Addis Ababa, Ethiopia (71.8%) [28]. The possible rational for this difference might be due to good personal hygiene practice and better life style modification like, good food handling was better among children in the current study than children in the above study. Moreover, better awareness was created about the problem among parents of children in the current study.

According to report of this study, the habit of eating raw vegetable was positively associated with intestinal parasites. This finding is consistent with finding of a study conducted in Thailand, Itabuna [29]. The possible explanation might be that study participants eat raw vegetables and fruits without washing their hands. This rational is enhanced by the fact that green vegetables and fruits are the main source of food in both study areas. Participants might handle and utilize vegetables with unhygienic matter without washing them. This study also indicated that the absence of household latrine was associated with intestinal parasites positively. Similar findings were reported from studies conducted in Senbete and Bete towns, North Shoa, Ethiopia and Tseda health center North West Ethiopia [26, 30]. This might be due to contamination of water and food with human waste during open field defecation. Besides, lack of awareness about importance of hand washing practice after defecation can lead to easily contaminate people through food. In addition, open field defection could increase parasite infection. Ethiopians, especially people in this study area, have a habit of eating raw meat. Hence, cattle meat is contaminated by people’s defecation during grassing. Shoe wearing practice was another important and statically significant factor in the current study. This result was consistent with studies conducted in North West Ethiopia [30]. This might be that parents of children were not informed about mode of transmission of parasites. Besides, children who walk on barefoot allowed penetration of their skin by the infective parasites. The current study also revealed that children live in large family size (more than eight) were positively associated with intestinal parasitic infections than children live in small family size. Similar finding reported from a study conducted in the Tigray region of Ethiopia [32]. This might be due to the fact that overcrowded family condition creates an increased opportunity for parasitic transmission. In addition, parents may not have time to care their child.

### Limitation of the study

This study had a number of strengths. Among these strengths, the community based nature of this study is representative of all children in the age group of 6 to 59 months which is essential in appropriate policy strategy for effective prevention of intestinal parasites. Regardless of its strengths, this study had few limitations. First, due to a cross-sectional design, it was difficult to examine any potential temporal relationships. Second, there might be a recalling bias for some study variables. Finally, children are only recruited within a single month, and there may be seasonal fluctuations of the prevalence of intestinal parasite in the study area.

## Conclusion

This study demonstrated that prevalence of intestinal parasites is still high among children of age 6 to 59 months. The absence of latrine facility, habit of eating raw vegetables, large family size and unable to wear shoes were factors positively associated with intestinal parasites. Policy makers should give priority for personal hygiene and environmental sanitation. In addition, awareness creation should be enhanced on life style modification for prevention of intestinal parasite infections.

## Data Availability

We have sent all the available data and we do not want to share the raw data as we are doing related study.

## Abbreviations

AOR: Adjusted Odds Ratio
CI: Confidence Interval
IRB: Institutional Review Board
SPSS: Statistical Package for Social Science
